# Integrating the ocean into the Planetary Boundaries framework: scientific gaps and pathways forward

**DOI:** 10.1093/nsr/nwag159

**Published:** 2026-03-13

**Authors:** Feifei Meng, Jingfang Fan

**Affiliations:** College of Ocean and Earth Sciences, Xiamen University, China; School of Systems Science, Beijing Normal University, China

## Abstract

The Planetary Boundaries (PB) framework, first introduced in 2009, has become one of the most influential conceptual tools in Earth system science for defining a ‘safe operating space for humanity’. It identifies nine critical Earth system processes, including climate change, biosphere integrity, and ocean acidification, and proposes quantitative boundaries beyond which humanity risks triggering irreversible environmental changes.

Despite the ocean covering 71% of Earth’s surface and playing a fundamental role in regulating climate, sequestering carbon, and maintaining global biogeochemical cycles, its representation within the PB framework remains notably limited. Only one boundary, ocean acidification, explicitly addresses marine systems, and even this focuses primarily on surface ocean chemistry. The ocean interior, deep-sea processes, subsurface biogeochemistry, large-scale circulation patterns, and marine functional diversity are largely absent.

This gap is not merely conceptual. As ocean industrialization accelerates, through deep-sea mining, high-seas fisheries, and the expanding ‘blue economy’, the need to understand how these activities impact planetary-scale stability becomes increasingly urgent. Meanwhile, the current governance landscape remains fragmented, with different ocean issues addressed under different regimes, lacking a coherent framework to guide decision-making.

Against this backdrop, this forum chaired by Professor Minhan Dai of Xiamen University brings together leading scholars in marine and Earth system science to address a pressing question: How can marine processes and ecosystems be effectively incorporated into the PB framework, and what would this mean for science, governance, and sustainability?

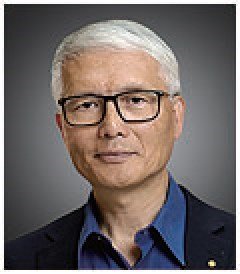

Deliang Chen

Professor, Department of Earth System Science, Tsinghua University, China

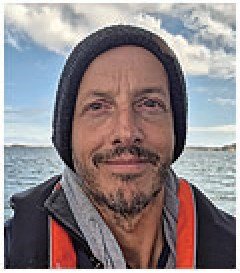

Sam Dupont

Senior Lecturer, Department of Biological & Environmental Sciences, University of Gothenburg, Sweden

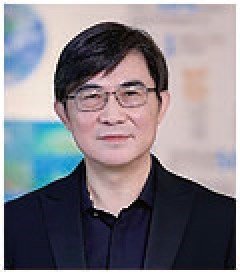

Minhan Dai (Chair)

Professor, College of Ocean and Earth Sciences, Xiamen University, China

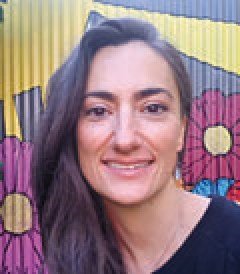

Ellycia Harrould-Kolieb

Lecturer, School of Agriculture, Food and Ecosystem Sciences, University of Melbourne, Australia

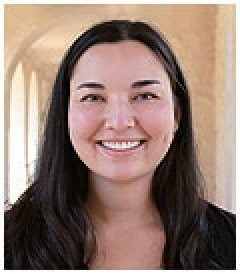

Ariel Pezner

Chancellor’s Research Fellow, University of Technology, Australia

## IDENTIFYING CRITICAL OMISSIONS IN THE CURRENT FRAMEWORK

**Dai:** The Planetary Boundaries (PB) framework sets safe operating limits for human activity, but oceans—covering two-thirds of the planet—are largely absent, a ‘major oversight’ limiting its usefulness. Given this, what are the key missing elements regarding the marine realm? Specifically, which crucial oceanic processes or components are inadequately represented, and how does their omission impact the framework’s effectiveness?

**Chen:** The PB framework was originally formulated from an Earth system science perspective at the global scale. It was not designed to address specific processes in detail. Rather, it aimed to identify overarching control variables that define a safe operating space for humanity.

However, effective conceptual frameworks including the PB framework must evolve as scientific understanding advances. Our knowledge of the oceans’ role in regulating the Earth system has grown substantially over the past decade. It is therefore time to think about incorporating critical yet previously underrepresented processes and phenomena, such as those associated with ocean dynamics.

These could include large-scale ocean circulation feedbacks—such as the Atlantic Meridional Overturning Circulation—as well as deoxygenation and other biogeochemical changes. Integrating such processes would strengthen the framework’s capacity to recognize the ocean not only as a buffer that absorbs anthropogenic forcing, but also as a potential amplifier of systemic change when critical thresholds are approached or crossed.

Our knowledge of the oceans' role in regulating the Earth system has grown substantially over the past decade. It is therefore time to think about incorporating critical yet previously underrepresented processes and phenomena, such as those associated with ocean dynamics.

—Deliang Chen

**Dupont:** It took me a long time to truly understand what the PB framework is. I remember seeing the presentation of Johan Röckström at the COP15 in Copenhagen, around the time the first article in *Nature* was published. I misunderstood the ‘safe operating space for humanity’ as a set of conditions that prevents or limits dramatic impacts of human activities on nature and society while it is linked to larger scale changes moving us out of the Holocene. My understanding is that humanity is already suffering and will increasingly suffer from environmental changes before a true shift toward a new geological era.

I was surprised by the prominent presence of ocean acidification as one of the original planetary boundaries. The ocean is currently impacted by a wide range of human impacts including over-exploitation of resources, habitat destruction, pollution, and global changes including ocean acidification. It was surprising that ocean acidification was the one most visible as it is currently not the main driver in the ocean. The other threats are ‘diluted’ in the other planetary boundary without explicit reference to the ocean.

It is very clear that the ocean is playing a key role in regulating the climate, carbon and other cycles, and is then highly relevant for the PB framework. The challenge will be to parametrize and quantify such impacts on such processes.

**Harrould-Kolieb:** We are seeing increased industrialization of the ocean realm and it is critically important that the profound and potentially irreversible pressures these activities exert on marine ecosystems are integrated into the PB framework. For example, there are ongoing negotiations around the potential for mining the deep sea in areas beyond national jurisdiction. If allowed to go ahead, this activity would inflict large-scale and severe disruption on deep sea ecosystems. Such direct anthropogenic forcing and habitat alteration are not well accounted for within the PB framework as it is currently conceptualized. One way of doing this could be through an expansion of the land-system change boundary to better account for ocean change. Indeed, it was proposed that this boundary be renamed ‘Earth surface change’ almost a decade ago to better incorporate impacts arising from large-scale anthropogenic alterations to the ocean floor, such as those anticipated from deep-sea mining, but we are yet to see this change adopted within the framework.

Another example can be seen in the biosphere integrity boundary, there is still much we don’t know about the state of ecosystems in areas beyond national jurisdiction, the area of the ocean that corresponds most closely to pelagic or open ocean ecosystems. Deep sea mining, high seas fisheries, marine heat waves, plastics, amongst other threats will all act together to undermine the integrity of open ocean systems, yet they are not well considered in the biosphere integrity boundary. Large-scale changes to the ocean floor and marine ecosystems need to be better accounted for in the PB framework. The introduction of a new ‘ocean integrity’ boundary would help to account for the large-scale changes that are occurring across the ocean and would acknowledge the importance of the ocean to planetary health and human survival.

The introduction of a new ‘ocean integrity’ boundary would help to account for the large-scale changes that are occurring across the ocean and would acknowledge the importance of the ocean to planetary health and human survival.

—Ellycia Harrould-Kolieb

**Pezner:** From my perspective, and that of many of my colleagues—Drs Erica Ferrer, Kevin Rose, and others from the Global Ocean Oxygen Network who have been leading this work—one of the most pressing missing pieces from the PB framework is the process of aquatic deoxygenation. In thinking of the main challenges that the ocean faces today, warming, acidification, and deoxygenation are three central and highly linked threats to the marine realm that can be directly tied to anthropogenic greenhouse gas emissions and climate change. As our planet warms, the oceans absorb more and more excess heat, driving ocean warming. This warming is leading to decreased solubility of oxygen in the oceans, increased respiration rates of marine life, and growing stratification—all of which drive the process of ocean deoxygenation. At the same time, the absorption of nearly a third of our carbon dioxide emissions by the ocean is driving shifts in oceanic carbonate chemistry, leading to ocean acidification. It is important to note that the processes of warming, acidification, and deoxygenation do not exist in isolation, but rather occur in complex combinations across the world’s oceans, with the potential for compound events that can have disastrous impacts on marine life and ecosystem function. However, only two of these threats are currently represented in the PB framework, as climate change and ocean acidification, so far leaving out deoxygenation.

Oxygen is a nearly universal requirement for life, both on land and underwater, and continued loss of oxygen stands to threaten the stability of the Earth system. Ocean deoxygenation is a global-scale process, with the capability of causing mass biological crises, and is projected to continue under climate change at similar rates as warming and acidification. Since the 1970s, the ocean has lost nearly 3% of its dissolved oxygen content, equivalent to the amount of oxygen that ∼320 million people breathe in a lifetime. Moreover, deoxygenation is not only affecting the marine realm, but also freshwater systems across the globe, with rates of oxygen loss since 1980 as high as 18.6% in the deeper parts of lakes and reservoirs, further highlighting the urgency and global reach of this key Earth system process across all bodies of water on this planet.

By excluding aquatic deoxygenation from the PB framework, we cannot accurately quantify, track, and communicate

By excluding aquatic deoxygenation from the PB framework, we cannot accurately quantify, track, and communicate the key threats to the Earth system under climate change. Aquatic deoxygenation provides a key link between atmospheric, terrestrial, and oceanic processes on our planet, and interacts with all other existing boundaries.

—Ariel Pezner

the key threats to the Earth system under climate change. Aquatic deoxygenation provides a key link between atmospheric, terrestrial, and oceanic processes on our planet, and interacts with all other existing boundaries. Despite its global importance, significantly less research has been done on the Earth system, biological, and societal impacts of deoxygenation compared to warming or acidification, and many key gaps in our knowledge remain to be filled. The omission of deoxygenation from the PB framework could delay urgently needed policy decisions, monetary investment, and research innovation in this field—ultimately at the cost to the ocean and the billions of people on this planet who rely on it.

## TOWARD AN INTEGRATED EARTH SYSTEM PERSPECTIVE

**Dai:** Building a fully holistic view of the Earth system requires bridging knowledge across terrestrial, atmospheric, and marine domains. Previous analyses noted that an historical terrestrial focus has ‘seriously hindered’ the efficacy of the PB framework. Efforts are now calling for a more integrated, holistic understanding of global environmental change that includes ocean processes. From your perspective, where are the biggest knowledge gaps that must be filled to achieve a truly holistic Earth system framework? Are there specific ocean–land–atmosphere linkages, feedbacks, or data deficiencies that stand out as priorities for research?

**Chen:** I do not think the PB framework reflects a historical terrestrial bias. It was conceived from a global Earth system science perspective. The real challenge, in my view, is not only about adding missing specific variables, but about insufficient understanding of how coupled processes propagate across spheres, across scales, and into human systems.

From my perspective, the most critical knowledge gaps lie in three interrelated areas: (1) coastal zone coupling as a core Earth system interface, (2) nonlinear cross-sphere cascades and tipping interactions, and (3) integrated long-term observations—particularly in ocean and coastal domains—to support truly coupled, predictive models.

First, the land–ocean interface, especially the coastal zone, remains a weak link in Earth system thinking. Coastal zones are where terrestrial hydrology, sediment and nutrient transport, ocean circulation, atmospheric extremes, ecosystems, and dense human activity converge and interact. Yet they are often treated as boundary regions rather than as core coupling domains. We need a more integrated understanding of coastal ‘critical zone’ processes—how sea-level rise, saltwater intrusion, storm surge, river regulation, eutrophication, wetland degradation, and coastal urbanization co-evolve, and how these processes feedback to climate regulation, carbon storage, and ecosystem services. Many global-scale risks indeed materialize through coastal amplification: sea-level rise, fisheries collapse, wetland loss, and salinity intrusion. Coastal systems are where planetary-scale changes translate into direct societal consequences.

Second, we still lack a robust science of nonlinear interactions and cascading risks across domains. The most consequential Earth system changes are unlikely to manifest as smooth linear trends; rather, they may emerge as threshold-like transitions and interacting cascades. Ocean dynamics are central in this regard. Circulation changes, marine heatwaves, deoxygenation, acidification, and ecosystem collapse can interact and propagate across physical, biogeochemical, ecological, and socioeconomic layers. We need frameworks that move beyond isolated tipping elements toward networked, multi-compartment dynamics, along with improved early-warning methodologies under deep uncertainty.

Third, the observational foundation for a holistic Earth system framework remains uneven, particularly for the ocean and coupled interface regions. Compared with atmospheric monitoring, our capacity to observe deep ocean processes, biogeochemical fluxes, ecosystem resilience, and coastal groundwater–surface water exchanges is still limited. Without sustained, integrated observation systems linking ocean physics, biogeochemistry, ecology, and human pressures, it is difficult to develop and validate predictive, fully coupled Earth system models.

Without sustained, integrated observation systems linking ocean physics, biogeochemistry, ecology, and human pressures, it is difficult to develop and validate predictive, fully coupled Earth system models.

—Deliang Chen

**Dupont:** My personal take on this is that the PB framework is more a communication tool rather than a true scientific challenge. My expertise is on ocean acidification and other global drivers. Parametrizing the impact of only one of these pressures on the earth system would require so many leaps of faith, that it is quite unrealistic. After 20 years of research, we are still struggling to parametrize at the local scale in places where research and data are well available.

I would argue that while the tool is useful and the objective honorable, there is a risk of wanting to be quantitative with such an approach. The concepts of tipping point, thresholds, boundaries as a single number for any driver is impractical and should be considered locally as a landscape taking into account local exposure, sensitivity, and the complex non-linear processes and interactions.

For sure, this complexity becomes simplified while moving from local to global scale. There is a moment in time when the crossing of multiple local tipping points will cascade toward a deviation from the Holocene, but our mechanistic understanding of the earth system is far too limited to clearly project when this will happen.

One may consider a humbler approach, highlighting the overwhelming evidence of changes all around the world, in all realms, as evidence that we are already on the red line.

The concepts of tipping point, thresholds, boundaries as a single number for any driver is impractical and should be considered locally as a landscape taking into account local exposure, sensitivity, and the complex non-linear processes and interactions.

—Sam Dupont

**Harrould-Kolieb:** We are starting to understand how truly interlinked the global ocean is, from brittle stars with ranges across entire ocean basins to fish abundance reliant on ocean connectivity. This suggests that activities in one location are likely to be felt far across the ocean. These telecouplings need to be better represented across the PB framework as they will have implications for the sensitivity of various boundaries. For example, there are studies that indicate that ocean-atmosphere coupling may offset some of the effects of deforestation on climate change. This should be considered in the land-system change boundary. However, implications of this coupling should also be considered across other boundaries, for instance, such offsetting increases upwelling in some areas, which in turn could increase ocean acidification. Thus, while this ocean-atmosphere coupling may strengthen the climate change boundary, it may simultaneously weaken that of ocean acidification.

But to be a truly holistic framework, we need to articulate this concept better across the social sciences and humanities in ways that open spaces for the integration of new ways of being with nature that aren’t predicated on commodification and exploitation but rather seek to reconnect humans with the natural world through reciprocal relations. We need greater understandings of how our current governance systems have not only not prevented transgression of the boundaries, but in many ways have actively allowed this to occur. Deeper acknowledgements of causation and equity need to be internalized in our governance systems.

The PB framework has highlighted that our current systems of governance and development are not sustainable and that we need a radical shift in the way that we relate to and interact with nature. In relation to the ocean, thinking of protection, rather than use, as the norm would help to maintain the integrity of ocean systems. Given that areas beyond national jurisdiction are governed collectively by all nations, they could be the first areas where this shift could be implemented. This could start with the banning of all fishing on the high seas and placing a moratorium on all deep sea mining. This would see great benefits for many of the planetary boundaries, including biosphere integrity, climate change, ocean acidification, biogeochemical flows, and land-system change, all of which are fundamentally supported by a healthy and flourishing ocean. Such bans would also recognize the inequity associated with exploitation of the ocean commons and be an important step in truly protecting this common heritage.

But to be a truly holistic framework, we need to articulate this concept better across the social sciences and humanities in ways that open spaces for the integration of new ways of being with nature that aren’t predicated on commodification and exploitation but rather seek to reconnect humans with the natural world through reciprocal relations.

—Ellycia Harrould-Kolieb

**Pezner:** I certainly agree that incorporating connections between marine, terrestrial, and atmospheric realms will be key in strengthening the impact and reach of the PB framework. The idea of better incorporating the connections of existing planetary boundaries to marine systems has been explored in more detail by others previously. For a new planetary boundaries of Aquatic Deoxygenation, the ocean–land–atmosphere linkages are clear. On a global scale, aquatic deoxygenation is driven by emissions of greenhouse gases to the atmosphere. At local scales, acute deoxygenation can also occur as a result of nutrient loading from land-based sources, often carried to the ocean along freshwater systems like rivers and streams. This nutrient loading can lead to periodic, seasonal, or even sustained deoxygenated conditions in coastal systems, leading to mass marine mortality events, human health impacts, and significant economic costs to local communities.

However, there still remain gaps in our knowledge and key priorities for research within the field of deoxygenation. High-frequency, high-quality, and spatially-resolved dissolved oxygen monitoring programs are a key need to advance our understanding of the broad impacts of oxygen loss. While other ocean metrics such as temperature and pH are regularly included in long-term monitoring programs across the oceans, records of dissolved oxygen, especially in highly dynamic coastal regions, are still scarce. It is also a high priority to create open databases of oxygen data to support transparency and data sharing. Additional data will allow us to monitor the progress of global deoxygenation, aid in constraining models of future trends, and facilitate investigation of the links between ocean oxygen change and ecosystem and organismal health. Lastly, we also need more data on the relevant thresholds of low oxygen for diverse marine species, especially in tropical systems like coral reefs where oxygen has been assumed to be plentiful. Together, these new data will help to identify control variable boundaries more accurately for the PB framework and track progress toward an unsafe space. Given the vast scale of the impacts of ocean deoxygenation on the global oceans, increased monitoring and data will be key pieces needed to achieve a truly holistic Earth system framework.

**Dai:** The PB framework is grounded in the idea that crossing certain thresholds could trigger critical transitions in the Earth system. However, ocean processes often exhibit complex nonlinear dynamics and may involve multiple regional tipping elements rather than a single global threshold. In your view, does the current PB framework adequately capture the risk of abrupt or cascading changes in the ocean system, or are there conceptual or operational gaps that remain?

**Chen:** The PB framework emerged from decades of Earth system research on nonlinear dynamics, resilience, and thresholds. To the question of whether the PB framework adequately addresses ocean tipping points and nonlinear behavior, my answer would be: conceptually, yes; operationally, not yet.

Conceptually, the framework acknowledges that the Earth system can undergo critical transitions. However, in practice, the representation of ocean dynamics—particularly their nonlinear, spatially heterogeneous, and multi-scale characteristics—remains insufficiently developed.

The ocean system, especially in coastal zones, often experiences gradual stress accumulation followed by nonlinear shifts. Coral reef collapse, deoxygenation, wetland degradation, and large-scale circulation slowdowns typically result from cumulative pressures rather than isolated shocks. These processes are rarely independent; they are interconnected and propagate across physical, biogeochemical, ecological, and socioeconomic layers.

The current PB framework adopts precautionary boundaries to avoid high-risk zones, which is both prudent and scientifically justified. Nevertheless, further development is needed in several directions:

Move beyond single scalar thresholds toward stability landscapes and resilience-based metrics.

Explicitly incorporate cascading cross-boundary interactions and networked tipping dynamics.

Recognize critical interface regions, such as coastal zones, as early-warning domains where planetary risks often manifest first.

In short, the next generation of PB thinking should integrate network-based tipping analysis and multi-sphere coupling, particularly at land–ocean interfaces. Doing so would enhance its operational capacity to address complex, nonlinear ocean behavior while preserving its strong scientific foundation.

## REFINING THE FRAMEWORK: INDICATORS, EQUITY, AND IMPLEMENTATION

**Dai:** Recently, scientists argued that aquatic deoxygenation should be adopted as a 10th planetary boundary, noting that many water bodies are approaching critical oxygen thresholds ‘at rates comparable to other planetary boundary processes’. In addition, the existing biosphere integrity boundary may not fully capture marine functional diversity—for instance, ocean food web changes or loss of key functional groups are not clearly reflected in the current metrics. In light of these points, do you think deoxygenation should be an additional planetary boundary, and why? Moreover, how would you suggest incorporating marine functional diversity into the PB framework so that changes in ocean ecosystems are properly accounted for?

**Pezner:** I wholeheartedly support the addition of deoxygenation as a planetary boundary. I was first introduced to this topic by Dr. Erica Ferrer after her presentation at the last GO2NE Oxygen Conference in 2022 and have been grateful to be a part of the group advocating for the inclusion of deoxygenation in the PB framework in recent years. Even without its interactions with warming and acidification, deoxygenation alone stands to threaten the stability of marine system function globally. As a result of deoxygenation, oxygen minimum zones across the oceans are expanding by 3%–8%, with 59%–80% of the oceans projected to be impacted by oxygen loss by 2050. Moreover, the IPCC identifies oxygen loss as one of the ‘five primary drivers of marine ecosystem change’ on our planet. Given that acidification and deoxygenation are two of these major ocean change processes, and are both driven by increasing atmospheric CO_2_ concentrations, I see no reason for ocean acidification to exist as a standalone boundary and not aquatic deoxygenation.

In terms of marine functional diversity, I support the inclusion of marine systems within the change in the biosphere integrity boundary. This integration may likely require a reassessment of the control variables used to define this particular boundary, as metrics like diversity of extinction rates do not truly capture changes in ecosystem function or trophic dynamics. Once this is determined, it could be useful to create sub-boundaries within the current control variables to account for individual changes in terrestrial, marine, and freshwater systems, so that impacts on these different components of the biosphere can be properly assessed and tracked.

**Dupont:** Oxygen, like temperature, is a very important threat to the marine environment. But I do not think that adding extra planetary boundaries is necessarily the way forward. The ocean is not explicitly represented enough in some of the other boundaries. Maybe a way forward is to give the ocean an equal space across boundaries and highlight pathways by which planetary boundaries are impacting the processes.

Importantly, I would also ensure that the different compartments and stressors are not considered in isolation but rather as interconnected leading to complex combined and cascading effects. While this is a feasible task conceptually, I still have reservations when it comes to serious and scientific parametrizations.

**Dai:** Some critics argue that the PB concept, while influential, is essentially a policy-oriented tool that oversimplifies complex Earth system science. A related critique is that focusing on multiple boundaries might spread political will too thin when perhaps more direct measures are needed. How do you respond to the claim that the PB framework is ‘not needed’ or not scientifically robust—that it’s merely a catchy concept for policymakers without a strong scientific basis? Do you agree with this skepticism?

**Chen:** I do not share the skepticism that the PB framework is scientifically weak. From my experience coordinating parts of the global scientific community during the Rio + 20 processes, I witnessed firsthand that the framework emerged from extensive discussion and engagement across a broad range of scientific disciplines and communities. It was not a narrow or isolated initiative, but rather a natural outcome of the development and maturation of Earth System Science.

Policy should be grounded in science, and science itself must evolve as new evidence and new challenges arise. Since its initial formulation in 2009, the PB framework has been updated and refined. It remains scientifically robust and highly valuable because it captures fundamental insights about Earth system dynamics relevant for policy.

For example, it reflects the recognition that the Earth system contains critical transitions—an understanding consistent with tipping-point theory and paleoclimate evidence. It has also proven useful as a risk governance framework under conditions of deep uncertainty. In my role coordinating scientific input to the Rio + 20 process, I observed that the PB framework attracted significant attention from policymakers worldwide and contributed meaningfully to discussions that helped shape the Sustainable Development Goals. Its precautionary boundaries were instrumental in articulating the need to reduce the probability of destabilizing regime shifts.

Moreover, the PB framework has been progressively ‘downscaled’ to regional contexts with some success. Its integrative architecture helps illuminate interactions across domains and translate Earth system science into a form usable for governance—without oversimplifying the underlying complexity.

Policy should be grounded in science, and science itself must evolve as new evidence and new challenges arise.

—Deliang Chen

**Harrould-Kolieb:** A planetary level framework is very important for bringing coherence across policy making and systemic integration across international law. Our governance system is highly fragmented with different issues governed under different regimes. High-level frameworks like the Planetary Boundaries framework can help guide the building of linkages across this fragmentation. The PB framework provides a higher order view of planetary systems and ideally their interlinkages thereby offering guidance as to where and how action is needed to prevent transgression of the boundaries.

However, as acknowledged earlier, the ocean is not fully integrated across this framework, which creates a blind spot for policy-making. Large-scale ocean activities will have impacts on the integrity of various boundaries; these need to be better understood across the framework to allow for better decision-making in the ocean space. When considering the increased industrialization of the ocean via development of the ‘blue economy,’ we need a better understanding of what impact this will have on our planetary system, we should be making these decisions in a holistic way, which is what a framework, such as the PB framework, offers.

A planetary level framework is very important for bringing coherence across policy making and systemic integration across international law.

—Ellycia Harrould-Kolieb

**Dai:** In order to determine whether humanity is approaching or crossing planetary boundaries, we rely on indicators for each boundary. When it comes to the ocean, there is an ongoing effort to develop a more comprehensive set of ocean indicators to track the health and stability of marine systems. Currently, the PB framework’s explicit ocean indicator is for acidification, and other ocean-related changes are indirectly included via boundaries like climate or biogeochemical flows. In your assessment, are the current ocean indicators sufficient to gauge whether we are nearing the ocean’s limits? What new metrics or observation systems might we need to better detect when we risk crossing a planetary boundary in the marine realm?

**Pezner:** I believe to most effectively capture if we are nearing the ocean’s limits, the ocean indicators within the PB framework should be expanded. While the aragonite saturation state can help to track ocean acidification, parameters like changes in ocean surface temperature, which could be added under the climate change boundary, or dissolved oxygen, which could be included with a new aquatic deoxygenation boundary, are key indicators that are so far not accounted for within the PB framework. Even with the ocean acidification boundary, there have been recent calls from the ocean acidification community to reassess the control variable boundary value, given high regional variation in acidification trends.

While many different groups have proposed lists of key ocean indicators over the years with some variation, key parameters such as sea surface temperature, pH, dissolved oxygen, and nutrient concentrations are consistently mentioned. Although some of these metrics are currently captured directly or indirectly in the current PB framework, a more ocean-focused reassessment of the framework could strengthen the representation of key ocean change parameters.

At the same time, new observation systems need to be established and expanded to ensure we are able to accurately detect when boundaries are crossed in the ocean. For example, monitoring of an aquatic deoxygenation boundary would require significant investment in additional long-term monitoring stations across the globe, both in the open ocean and along coastlines. For all ocean indicators, we need more observations from below the ocean’s surface. Remote sensing technologies have made it easy to track some of these ocean indicators across wide swaths of the ocean, but only at the surface. While the effects of climate change are being reflected relatively quickly in the surface ocean, the true impacts of these changes in the interior and deep ocean will not be felt for decades. Advances in ocean observing technologies such as ARGO profiling floats and gliders have helped us start to chip away at consistently monitoring interior ocean properties and trends, but the current ARGO fleet can only cover so much of our vast ocean. Moreover, only a subset of these ARGO floats are capable of measuring biogeochemical parameters like oxygen, pH, nitrate, and chlorophyll-a that would be key for monitoring planetary boundaries related to deoxygenation and modification of biogeochemical flows. Increasing our ocean monitoring capabilities will be key to better detecting when we risk crossing a planetary boundary in the marine realm, both at the surface and at depth.

Increasing our ocean monitoring capabilities will be key to better detecting when we risk crossing a planetary boundary in the marine realm, both at the surface and at depth.

—Ariel Pezner

## CONSENSUS AND FORWARD OUTLOOK

**Dai:** The conversation around planetary boundaries is evolving to include not just what is safe for Earth’s systems, but also what is just for humanity. In your view, how should we define safe and just boundaries in the Earth system? And importantly, who should be involved in setting these thresholds? Should it be solely the domain of scientists, or a broader dialogue including policymakers, ethicists, and representatives of vulnerable communities? How can we ensure the process of defining safe and just boundaries is both scientifically credible and democratically legitimate?

**Harrould-Kolieb:** Ultimately the planetary boundaries are about maintaining Earth system functionality and maintaining Holocene-like conditions is thought to protect the ‘safe operating space’ for humanity. Transgressing the boundaries will push us beyond this safe space. However, while this may be true for humanity as a whole, there will be many people that will suffer, therefore not everyone is ‘safe’ and thus, maintenance of the planetary boundaries must be coupled with justice for all within the safe operating space.

For an equitable and just transition to a future where we live within the planetary boundaries our provisioning systems must be fundamentally restructured. Some believe that this can be done while remaining within our current structural paradigm of neoliberal capitalism and its variants. However, this paradigm has allowed for the extreme exploitation of both people and nature for the benefit of a few. For a truly ‘safe and just’ future we will need a radical shift away from this profit-driven model, not just a ‘greening’ of it. Alternative metrics to Gross Domestic Product (GDP), such as happiness, as a way of measuring country performance is one step in this process. As is the acknowledgement of the right of nature to exist and flourish, regardless of the benefits it provides to humans. These efforts are taking root across the globe and will help move us to a new normal where the extreme wealth of a few does not need to come at the cost of the exploitation of others.

Shifting the paradigm will require a redefinition of what it means to live a good life, including the elimination of endless cycles of consumption and the pursuit of affluence. We need to shift society away from enabling the most wealthy and privileged, to lifting up the marginalized and impoverished. Without righting existing structural inequalities, we will not be able to secure a safe and just future for all. The PB framework provides us with an understanding of the window in which we can operate for a safe future; this can be achieved in both just and unjust ways. In order for it to be just for all, we need to collectively decide that this is the future we want. To implement this, we need governance systems that are responsive to this desire. We also need to know where we are heading and for this, we need social, political, and legal imaginaries that offer us visions of a safe and just future world.

## Notes

Feifei Meng is an Engineer at the College of Ocean and Earth Sciences, Xiamen University, China

Jingfang Fan is a Professor at the School of Systems Science, Beijing Normal University, China.

